# Benefits and harms of gastric suction or lavage at birth for gastrointestinal outcomes: A systematic review and meta-analysis

**DOI:** 10.1371/journal.pone.0288398

**Published:** 2023-07-13

**Authors:** Nanthida Phattraprayoon, Teerapat Ungtrakul, Mingkwan Na Takuathung

**Affiliations:** 1 Princess Srisavangavadhana College of Medicine, Chulabhorn Royal Academy, Bangkok, Thailand; 2 Department of Pharmacology, Faculty of Medicine Chiang Mai University, Chiangmai, Thailand; 3 Clinical Research Center for Food and Herbal Product Trials and Development (CR‐FAH), Faculty of Medicine, Chiang Mai University, Chiang Mai, Thailand; Qatar University, QATAR

## Abstract

The benefits of routine gastric suctioning or lavage in neonates remain uncertain, despite the common practice worldwide. To investigate the potential advantages and harms, we conducted a systematic review and meta-analysis of randomized controlled trials (RCTs) examining the effects of these procedures in healthy or meconium-stained neonates at birth. We systematically searched PubMed, Scopus, Embase, Ovid, and the Cochrane Library databases from inception to February 9, 2023. We included only RCTs assessing the outcomes of gastric suction or lavage in neonates at birth. We calculated risk ratio (RR) and weighted mean differences with 95% confidence intervals (CIs) using a random-effects model. The primary outcomes were gastrointestinal symptoms including vomiting, retching, feeding intolerance, and secondary aspiration. The secondary outcomes included time to initiation of breastfeeding and potential adverse procedure-related events. Twelve RCTs with a total of 4,122 neonates were analyzed. All the studies compared neonates who received gastric suction or lavage with those who received usual care. Gastrointestinal symptoms were significantly reduced in neonates receiving gastric suction or gastric lavage compared with the control group (RR, 0.75; 95% CI, 0.63–0.89). Gastric lavage was beneficial for infants with meconium-stained amniotic fluid (RR 0.71; 95% CI, 0.60–0.84), while gastric suction had no significant benefit in reducing gastrointestinal symptoms in infants without meconium-stained amniotic fluid (RR 0.91; 95% CI, 0.61–1.37). Our findings suggest that gastric suction or lavage may reduce gastrointestinal symptoms in neonates; however, these procedures may only benefit infants born with meconium-stained amniotic fluid. Vigorous newborns without meconium-stained amniotic fluid may not benefit from these procedures. Furthermore, gastric suction may lead to adverse outcomes such as apnea and bradycardia.

**Registration**: This study was registered in the PROSPERO International prospective register of systematic reviews in health and social care (CRD42023247780).

## Introduction

Gastric suction remains a common procedure performed in neonates at birth or during the first few hours of life in many regions of the world [1, 2]. Gastric suction involves suctioning through the oral cavity and the advancement of nasogastric tubes through the stomach to aspirate gastric contents that the infant may have ingested during the perinatal period [3]. In addition to suctioning through the oral cavity and stomach, some infants also receive irrigation of the gastric contents using normal saline, referred to as gastric lavage [4]. Gastric suction is performed in healthy newborns to eliminate amniotic fluid or gastric contents with the aim of reducing gastrointestinal (GI) symptoms such as nausea, vomiting, and aspiration to the airway [1, 2, 5]. In infants with meconium-stained amniotic fluid (MSAF), gastric lavage is used to prevent symptoms of GI discomfort such as vomiting, retching, feeding intolerance, and secondary meconium aspiration syndrome [6, 7]. Meconium may cause inflammation and chemical damage to the lungs [8], as well as GI tract disturbances [7, 9]. However, the benefits of gastric suction or gastric lavage on GI outcomes are still unclear, and there is a need to understand if gastric suction or gastric lavage is a beneficial routine practice in neonates.

Currently, three randomized controlled trials (RCTs) have evaluated the benefits of routine gastric suction in healthy neonates [1, 2, 5] and nine RCTs have investigated the use of gastric lavage in vigorous infants with MSAF [7, 10–17], while a previous meta-analysis focused on gastric lavage in MSAF [18, 19]. We therefore conducted a systematic review and meta-analysis to evaluate the benefits and harms of performing routine gastric suctioning or gastric lavage in vigorous neonates.

## Materials and methods

### Study protocol, registration, and ethical approval

This systematic review and meta-analysis followed the Preferred Reporting Items for Systematic Reviews and Meta-Analyses (PRISMA) 2020 guidelines [20, 21]. The research protocol was registered in the PROSPERO international prospective register of systematic reviews in health and social care (CRD42023247780). The Research Ethics Committee of the Princess Srisavangavadhana College of Medicine, Chulabhorn Royal Academy, granted an exempt research determination for this study protocol (No. 108/2564).

### Data sources and search strategy

We performed a comprehensive and systematic search of the PubMed, Scopus, Embase, Ovid, and Cochrane Library databases from inception to February 9, 2023, using the search terms (newborn OR neonate) AND gastric AND (suction OR aspiration OR decompression OR lavage OR wash OR irrigation). Publication reference lists were also searched manually for potentially relevant studies, with no language restriction. Records identified from the database searches were imported into Zotero software and duplicates were removed. The titles and abstracts were screened, and the full texts of articles that contained information relevant to the scope of the study were retrieved.

### Eligibility criteria

Articles were considered eligible for inclusion if they were randomized controlled trials (RCTs) or quasi-RCTs involving newborn participants who received gastric suction or gastric lavage, and if the intervention outcomes were reported. Cohort studies, case-control studies, uncontrolled trials, case series, letters, editorials, protocols, commentaries, and expert opinions were excluded from this systematic review.

The participants in the included studies were vigorous newborns, born with or without MSAF. Babies who were born without respiratory symptoms or the need for cardiorespiratory support at delivery were considered to be vigorous newborns. The interventions were gastric suction or gastric lavage during the first few hours of life. The control was usual care or standard care. The outcomes were GI symptoms (vomiting, retching, feeding intolerance), secondary aspiration, time to initiate breastfeeding, and adverse events.

Vomiting was defined as any episode of expulsion of gastric contents with effort occurring sometime after feeding, and which contained altered milk, meconium, or bile [13, 15]. Regurgitation was defined as effortless expulsion of milk during or immediately after feeding [13, 15]. Retching was defined as attempted vomiting without expulsion of any gastric contents [13]. GI symptoms, GI disturbance, or feeding problems were defined as vomiting, retching, regurgitation, abdominal distension, or gastric residual [1, 2, 5, 7, 11, 13, 14, 17]. Feeding intolerance was defined as more than two episodes of vomiting in any 4-h period or more than three in 24 h, and/or abdominal distension (increase in abdominal girth by 2 cm from baseline), and/or gastric residual volume >2 mL undigested milk or bilious color [10, 12].

### Study selection

Two researchers (N.P. and M.N.) independently screened the scientific literature for relevant data based on the inclusion criteria, study design, methodology, outcome parameters, and quality of included studies. Disagreements were resolved through discussion with a third researcher (T.U.).

### Data extraction and quality assessment

The following data and outcomes were extracted from the included RCTs: first author, year of publication, study design, country of origin, participant characteristics, type of intervention, GI outcomes such as vomiting and retching, and time of breastfeeding initiation. The investigators of the studies were contacted via email regarding any missing or unreported data, or to obtain additional information.

The study evaluated five areas of bias using the RoB2 tool, as the most common method for assessing bias in RCTs: bias from the randomization process, deviations from intended interventions, missing outcome data, outcome measurements, and selection of reported results [22]. The researchers classified each RCT as having low or high risk of bias or having some concerns, based on the RoB2 algorithm. The results of the RoB2 assessment were displayed visually using an R package and the Shiny web app, Risk-of-bias VISualization (robvis) [23].

### Data synthesis and statistical analysis

To determine the benefits of gastric suction or gastric lavage in neonates, we conducted a systematic analysis using a random-effects model. This model was chosen due to the observed heterogeneity among the included studies, which encompassed variations in study designs, populations, interventions, and outcomes. The random-effects model considers both within-study and between-study variability, providing a more conservative and robust estimate of the overall effect. For categorical variables, we calculated risk ratio (RRs), while for continuous outcome, we estimated weighted mean differences (MDs). Each pooled estimate was accompanied by a 95% confidence interval (CI) to assess the precision of the results. To explore the sources of heterogeneity, we performed intervention- or patient-based subgroup analysis. We assessed the statistical heterogeneity using Q-statistic and *I*^*2*^ tests, with *I*^*2*^ values of 25%, 50%, and 75% indicating low, moderate, and high heterogeneity, respectively. The level of statistical significance was set at 0.05. All meta-analyses were conducted using the Review Manager 5.4.1 software [24]. To investigate the possibility of small-study effects and publication bias, we visually examined funnel plots that plotted the effect size of each individual RCT on the horizontal axis against its standard error on the vertical axis [25]. Additionally, for meta-analyses comprising ten or more studies, we conducted the Egger’s test [26]. These analyses were performed using the Comprehensive Meta-Analysis Version 4 [27, 28].

The level of evidentiary certainty for each outcome was classified as high, moderate, low, or very low using the GRADE method [29, 30], with risk of bias [31], inconsistency [32], indirectness [33], imprecision [34], and publication bias [35] as the five degrading criteria. Summary of findings tables were generated using the GRADEpro Guideline Development Tool (http://gradepro.org).

## Results

### Search results

**Fig 1:**
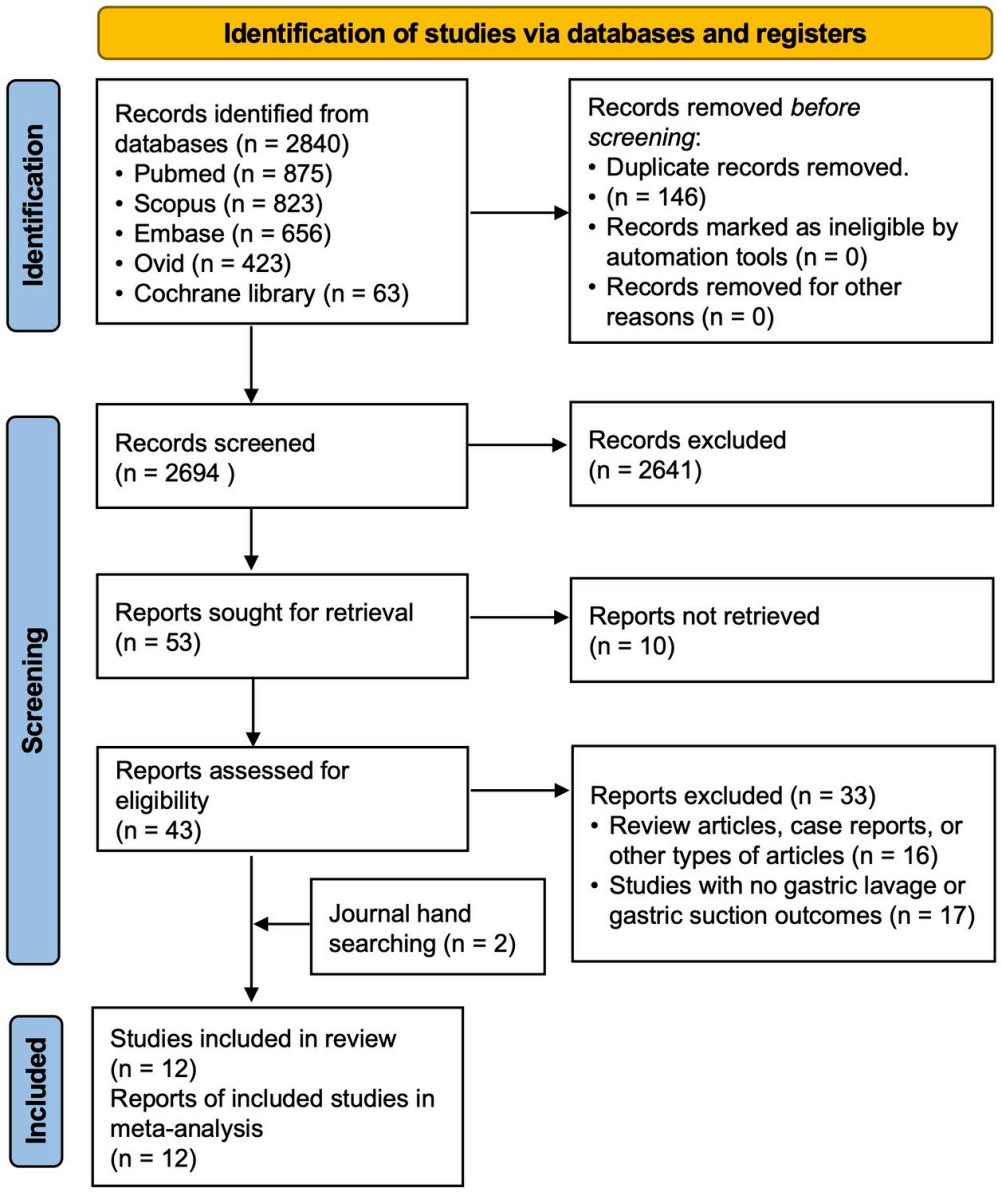
PRISMA flow diagram. Study selection for the systematic review and meta-analysis.

The database search yielded 2840 citations (Fig 1). After screening the titles and abstracts, 43 full texts were screened, 10 studies fulfilled the inclusion criteria, and two additional records were retrieved from other sources. Twelve studies [1, 2, 5, 7, 10–17] were finally included in our systematic review and meta-analysis. No further publications were obtained from the reference lists of the included studies.

### Study characteristics

The 12 RCTs [1, 2, 5, 7, 10–17] involved 4,122 patients, including 2,000 who received gastric suction or gastric lavage and 2,122 who did not receive these procedures. Each RCT compared gastric suction or gastric lavage with the standard of care. Three RCTs [1, 2, 5] provided gastric suction for infants without MSAF and nine studies [7, 10–17] provided gastric lavage for infants with MSAF. The included studies and maternal and infant characteristics are summarized in Table 1 and S1 Table, respectively. Seven investigations were conducted in India [10–13, 15–17] and one each was conducted in Nepal [14], Saudi Arabia [7], Mexico [2], Sweden [5], and Turkey [1]. All the included studies were published between 1987 and 2018. The objectives of the included studies were comparable, but the terms used by the authors varied. The primary aims of the included studies related to GI symptoms. Most studies used GI outcomes including vomiting, retching, or regurgitation as the main outcomes [1, 2, 5, 7, 10, 11, 13–15, 17]. The terms related to GI symptoms including vomiting, retching, and regurgitation were used in all previous studies [1, 2, 5, 7, 10–17], but were defined using different terminology. Some studies also employed the terms feeding intolerance, feeding problem, and feeding difficulty [10, 12, 15, 16] with GI symptoms as part of the definition. Increased abdominal girth or feeding residual were also symptoms of feeding intolerance [10, 12, 15, 16]. Most of the included RCTs had some risk of bias, and one trial showed a high risk of bias (S1 Fig).

**Table 1:** Characteristics of included studies.

Study Year	Type of study	Location	Inclusion criteria	Exclusion criteria	Randomization	Study period	Control	Intervention
**Widström 1987 [5]**	RCT; single blinded	Sweden	- Full-term infants with birth weight 3–kg	Pregnancy without complications	Blocks by sex	NR	No gastric suction	Gastric suction at birth
**Narchi & Kulaylat 1999 [7]**	RCT; non-blinded	Saudi Arabia	Neonates with MSAF; GA ≥34 weeks	Birth asphyxia Hemodynamic instability Respiratory distress Severe pallor Major congenital anomalies	Odd and even days	2 years	No gastric lavage	Gastric lavage within 1 h after birth and before first feed
**Cuello-Garcia et al. 2005** [2]	RCT; single-blinded	Mexico	Neonates with birth weight 2500–4000 g; Apgar score at 5, 10 mins ≥8	Neonates with MSAF Need for cardiopulmonary resuscitation Contraindications for feeding Major congenital anomalies	Blocks of six and program	5 months; May to Sep 2004	No gastric lavage	Gastric lavage within 30 minutes after birth
**Kiremitci et al. 2011** [1]	RCT; blinded	Turkey	GA >37 weeks; birth weight 2500–4200 g	Multiple pregnancy GA ≤37 weeks Maternal morbidity Early ROM MSAF Vaginal delivery with non-cephalic presentation Fetal distress	NR	5 months; Sep 2008 to May 2009	No gastric suction	Gastric suction at birth
**Ameta et al. 2013** [10]	RCT; non-blinded	India	Neonates with MSAF (vigorous); GA ≥34 weeks; birth weight ≥1800 g	Non-vigorous babies Major congenital anomalies Respiratory distress with Downes score ≥3 Required cardiopulmonary resuscitation at birth	Computer-generated random numbers	11 months; Dec 2009 to Oct 2010	No gastric lavage	Gastric lavage at birth
**Singh et al. 2013** [11]	RCT; blinded	India	Neonates with MSAF (vigorous); GA ≥36 weeks	Apgar score < 5 at 5 min Hemodynamic instability Respiratory distress Major congenital anomalies	Blocks	NR	No gastric lavage	Gastric lavage soon after birth
**Garg et al. 2014** [12]	RCT; non-blinded	India	Neonates with MSAF (vigorous);- GA ≥34 weeks; birth weight ≥1800 g	HIE Major congenital malformations Respiratory distress with Downes score >3 Required cardiopulmonary resuscitation at birth	Computer-generated random numbers	12 months; Aug 2011 to Jul 2012	No gastric lavage	Gastric lavage within 30 min after birth
**Sharma et al. 2014** [13]	RCT; blinded one way	India	Neonates with MSAF (vigorous); GA ≥34 weeks	Major congenital anomalies Respiratory distress Requiring oxygen	Computer-generated random numbers	6 months; Mar to Sep 2011	No gastric lavage	Gastric lavage at birth
**Shah et al. 2015** [14]	RCT; non-blinded	Nepal	Neonates with MSAF (vigorous); GA ≥34 weeks; birth weight ≥1800 g	Major congenital anomalies Respiratory distress Requiring oxygen	Computer-generated random numbers	12 months; Jul 2013 to Jun 2014	No gastric lavage	Gastric lavage at birth
**Kumar et al. 2017** [15]	RCT; non-blinded	India	Neonates with MSAF (vigorous); GA >34 weeks; birth weight >1800 g	Congenital malformations Required resuscitation Respiratory distress	Computer-generated random numbers	14 months; Jul 2015 to Sep 2016	No gastric lavage	Gastric lavage at birth
**Gidaganti et al. 2018** [16]	RCT; non-blinded	India	Neonates with MSAF (vigorous);—GA ≥34 weeks	Gross congenital anomalies Mothers with suspected chorioamnionitis Mothers who receiving methyldopa during pregnancy	Computer-generated random numbers	NR	No gastric lavage	Gastric lavage at birth
**Yadav et al. 2018** [17]	RCT; blinded	India	Neonates with MSAF; GA ≥34 weeks	Requiring NICU admission Major congenital malformations	Computer-generated random numbers	19 months; Feb 2015 to Aug 2016	No gastric lavage	Gastric lavage at birth

**Abbreviations:** GA, gestational age; HIE, hypoxic ischemic encephalopathy; MSAF, meconium-stained amniotic fluid; NR, no report; NICU, neonatal intensive care unit; RCT, randomized controlled trial; ROM, rupture of membrane

### Risk-of-bias assessment

The results of the risk of bias assessment for the included RCTs are summarized in S1 Fig In 10 studies [2, 5, 7, 10, 12–17], the allocation sequence was generated, and seven of 12 utilized concealed allocation [2, 10, 12–14, 16, 17]. There were no double-blind RCTs among the studies. Loss to follow-up was acceptable [1, 2, 5, 7, 10–17], but selective reporting of outcomes showed some concern [1, 2, 5, 7, 10–17].

### Effects of gastric suction or gastric lavage on clinical outcomes

**Fig 2:**
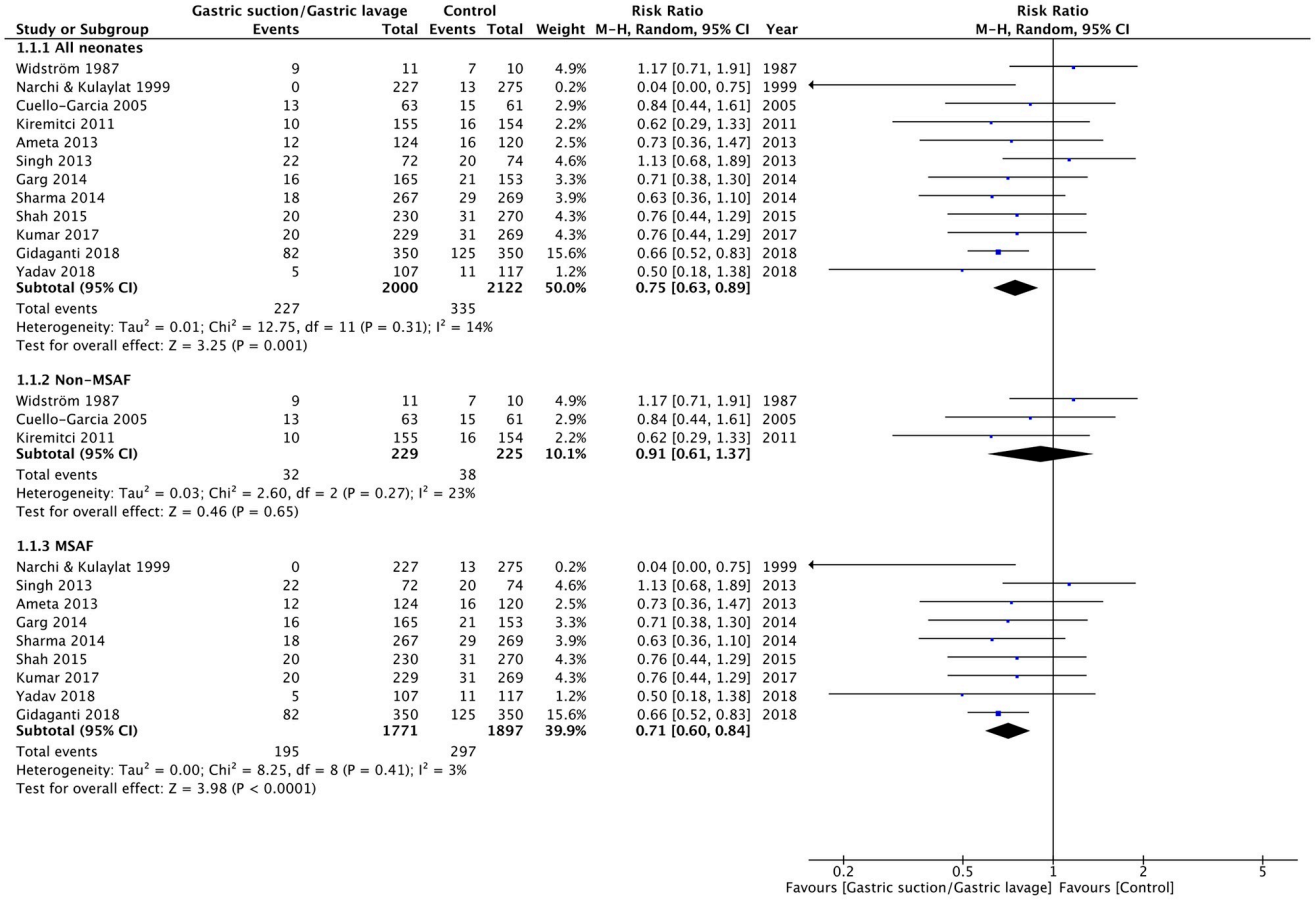
Forest plots. Effects of gastric suction or gastric lavage on gastrointestinal symptoms in all neonates, non-meconium-stained amniotic fluid (non-MSAF) neonates, and MSAF neonates.

**Fig 3:**
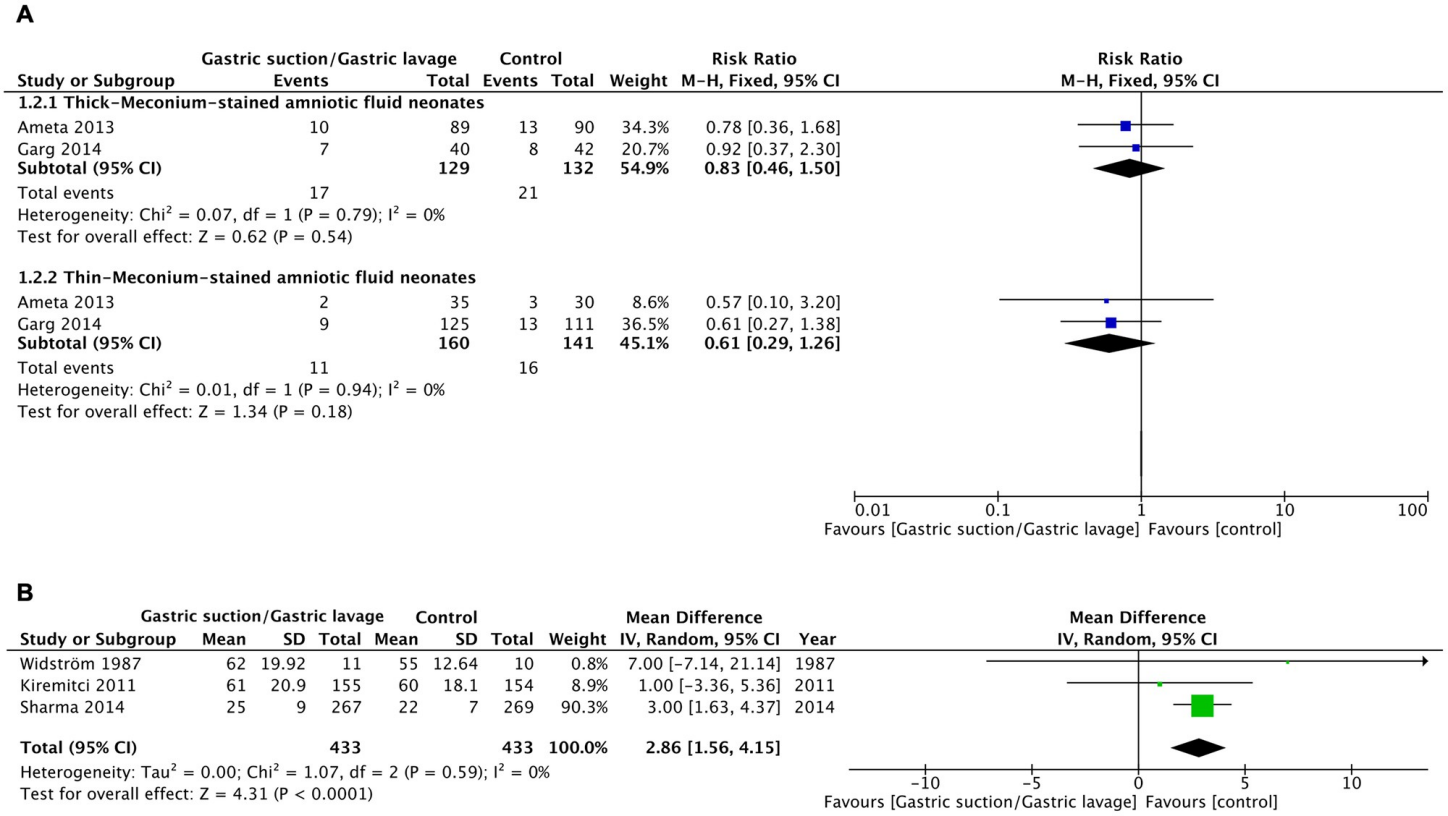
Forest plots of effects of gastric suction or gastric lavage on clinical outcomes. (A) Feeding intolerance in thick- and thin-meconium-stained amniotic fluid (MSAF) neonates. (B) Time to initiate breastfeeding (min).

The benefits of gastric suction or gastric lavage on clinical outcomes are depicted in Figs 2 and 3, Table 2, and S2 Table.

**Table 2:** GRADE summary of findings: Benefits of gastric suction or gastric lavage on clinical outcomes.

Patient or population: Vigorous infants Intervention: Gastric suction or gastric lavage Comparison: No treatment or usual care											
Study design	No. of studies	Certainty assessment					No. of participants		Effect		
		Risk of bias	Inconsistency	Indirectness	Imprecision	Other considerations	Gastric suction or gastric lavage	Placebo	Estimation of absolute effects		Certainty
									Risk (95% CI)	Absolute (95% CI)	
**Benefits of gastric suction or gastric lavage in neonates**											
**Gastrointestinal symptoms in all newborns**											
RCT	12	Serious	Not serious	Not serious	Not serious	Not serious	227/2000 (11.3%)	335/2122 (15.8%)	**RR 0.75** (0.63–0.89)	**39 fewer per 1,000** (58–17 fewer)	⨁⨁⨁⊝ Moderate
**Gastrointestinal symptoms in MSAF infants**											
RCT	9	Serious	Not serious	Not serious	Not serious	None	195/1771 (11.0%)	297/1897 (15.7%)	**RR 0.71** (0.60–0.84)	**45 fewer per 1,000** (63–25 fewer)	⨁⨁⨁⊝ Moderate
**Gastrointestinal symptoms in non-MSAF infants**											
RCT	3	Serious	Not serious	Not serious	Serious	None	32/229 (14.0%)	38/225 (16.9%)	**RR 0.91** (0.61–1.37)	**15 fewer per 1,000** (66 fewer to 62 more)	⨁⨁⊝⊝ Low
**Feeding intolerance in thick-MSAF infants**											
RCT	2	Serious	Not serious	Not serious	Serious	None	17/129 (13.2%)	21/132 (15.9%)	**RR 0.83** (0.46–1.50)	**27 fewer per 1,000** (86 fewer to 80 more)	⨁⨁⊝⊝ Low
**Feeding in tolerance in thin-MSAF infants**											
RCT	2	Serious	Not serious	Not serious	Serious	None	11/160 (6.9%)	16/141 (11.3%)	**RR 0.61** (0.29–1.26)	**44 fewer per 1,000** (81 fewer to 30 more)	⨁⨁⊝⊝ Low
**Time to initiate breastfeeding (min)**											
RCT	3	Serious	Not serious	Not serious	Serious	None	433	433	-	MD **2.86 higher** (1.56–4.15 higher)	⨁⨁⊝⊝ Low

**Abbreviations:** MSAF, meconium-stained amniotic fluid; CI, confidence interval; MD, mean difference; RCT, randomized controlled trial; RR, risk ratio

#### GI symptoms

Twelve RCTs provided evidence regarding GI symptoms [1, 2, 5, 7, 10–17]. Gastric suction or gastric lavage reduced GI symptoms, with moderate-quality evidence (RR 0.75; 95% CI, 0.63–0.89). Subgroup analysis was conducted according to MSAF. In nine studies, infants with MSAF benefited from gastric lavage (RR 0.71; 95% CI, 0.60–0.84) [7, 10–17] with moderate-quality evidence, while three RCTs [1, 2, 5] that included non-MSAF infants found no significant benefit of gastric suction in reducing GI symptoms (RR 0.91; 95% CI, 0.61–1.37), with low-quality evidence. There was no evidence of the influence of small-study effects, including publication bias, as the funnel plot in the meta-analyses was symmetrical (S2 Fig).

#### Feeding intolerance in infants with thick and thin MSAF

Two studies [10, 12] with low-quality evidence demonstrated that gastric lavage did not improve feeding in MSAF infants with either thin or thick meconium (RR 0.61; 95% CI 0.29–1.26; RR 0.83; 95% CI 0.46–1.50, respectively).

#### Secondary aspiration

Only one in seven studies of non-MASF infants showed secondary aspiration, but this finding was not significant (RR 0.63; 95% CI 0.21–1.89]) [16]. No pooled analysis was carried out because only a single study demonstrated secondary aspiration. None of the infants in the remaining six non-MASF studies had secondary aspiration, regardless of the use of gastric lavage.

#### Time to initiate breastfeeding

Three randomized controlled trials [1, 5, 13] that analyzed the timing of breastfeeding initiation in vigorous neonates revealed a statistically significant delay in breastfeeding initiation following gastric suction or lavage (MD 2.86 min; 95% CI 1.56–4.15) with low-quality evidence (Fig 3, Table 2, S2 Table).

#### Adverse reactions

All nine studies of gastric lavage in MSAF infants showed that none of the infants in either group experienced apnea, bradycardia, or localized trauma [7, 10–17]. However, two studies that provided gastric suction found bradycardia in one infant [5] and apnea in one infant [1], respectively.

## Discussion

Gastric suction or gastric lavage is used to remove the gastric contents in neonates by suctioning it out or using a saline solution to wash it out, either at birth or during first few hours of life. The procedures are considered to prevent aspiration and reduce GI symptoms such as vomiting, retching, and regurgitation, as well as feeding difficulties caused by foreign material in the stomach, which stimulates these symptoms [9]. For babies born with MSAF, there is a concern that GI symptoms might lead to feeding difficulties and aspiration, and some practitioners therefore perform additional procedures such as gastric lavage, with the aim of improving feeding, decreasing the aspiration risk, and reducing GI symptoms. However, there is currently no evidence to support this practice [7]. Therefore, this systematic review and meta-analysis aimed to evaluate the benefits and harms of performing routine gastric suctioning or gastric lavage in vigorous neonates.

The current meta-analysis found that gastric suction or gastric lavage was beneficial in newborns overall. Based on subgroup analysis however, we found that healthy newborns received no benefits from gastric suction performed at birth, while vigorous infants with MASF benefited from gastric lavage in the first few hours, resulting in reduced GI symptoms such as vomiting or retching compared with similar infants without gastric lavage. The consistency of the meconium was initially assumed to be a crucial factor, but analysis of two trials [10, 12] failed to demonstrate if thick or thin meconium affected the benefits of gastric lavage in vigorous MSAF infants. The consistency of meconium may thus not be a relevant factor in terms of deciding whether or not to perform gastric lavage to reduce feeding intolerance. One concern when performing these procedures is the effect on the time to initiate breastfeeding. Three randomized controlled trials [1, 5, 13] revealed that the time to begin breastfeeding was statistically significantly delayed by 1–4 min in neonates who received these procedures (MD 2.86 min). Though this difference was statistically significant, it had no clinical significance, suggesting that these procedures may have no impact on the initiation of breastfeeding in clinical practice.

Previous systematic reviews and meta-analyses compared gastric lavage in vigorous MSAF infants with feeding intolerance, and showed that gastric lavage decreased feeding intolerance, with low-moderate quality evidence [18, 19]. Gastric suction may also cause bradycardia and apnea [1, 5], though there was no report of apnea, bradycardia, or local tissue trauma in the baby who received gastric lavage. Anand et al. demonstrated that noxious stimulation caused by gastric suction at birth may lead to an increased prevalence of functional intestinal disorders in later life [36].

The current study determined if gastric suction or gastric lavage was beneficial in vigorous neonates, regardless of MSAF status. Both procedures were used to clear secretion and gastric contents. The results revealed that gastric suction had no effect on GI symptoms in neonates without MSAF, while gastric lavage with meconium removal significantly reduced GI discomfort in MSAF neonates. Aspirated meconium may act as a chemical irritant to the GI tract and its aspiration into the lungs may also injure the lung tissues. Chemical irritation is thus one mechanism of meconium aspiration syndrome. This may explain why neonates who ingest meconium may experience nausea, vomiting, and retching; however, researchers remain skeptical that the amount of content swallowed by babies affects their symptoms, and this aspect was not reported in the included studies. Gastric lavage could benefit in the removal of more gastric content, especially thick and viscous content. However, this procedure is more time-consuming and is only used in specific circumstances. Generally, a healthcare provider will perform gastric suction at birth along with clearing the airway at birth. A baby without MSAF may not have significant or irritated contents that cause gastrointestinal symptoms. The results of gastric suction in non-MSAF group were unremarkable. However, because MSAF is more turbid than typical amniotic fluid, an infant with MSAF who ingests MSAF may have experienced gastrointestinal symptoms. The removal of this GI content via gastric lavage may help decrease gastrointestinal symptoms. The current Neonatal Resuscitation Program (NRP) 2020 does not recommend routine endotracheal suctioning of vigorous or non-vigorous infants with MSAF, but there is no guidance regarding gastric clearing in MSAF neonates. NRP suggests placing an orogastric tube in baby who received prolonged positive pressure ventilation or continuous positive pressure ventilation for venting the gas and removing the gastric contents [37].

In this meta-analysis, we analyzed the advantages of gastric suction and gastric lavage in vigorous neonates. The strength of this study is that this study we included all neonates with and without MSAF, in a total of 12 studies. In addition, the studies were conducted in diverse ethnic groups, including low- and high-resource settings, the outcomes mostly represented pooled estimates with a low degree of heterogeneity. This suggests that the effect of the intervention may be consistent across different populations and settings, and that the intervention could be a viable option for a broad range of patients. However, the current study had some limitations. The included studies were conducted in different regions of the world which may thus have varying implications for the care of newborns, particularly in light of recent advances in perinatal treatment. Notably, one of the trials [5] was conducted three decades ago, since when the approach to perinatal care for infants born with MSAF has evolved. Specifically, routine endotracheal suctioning of MSAF infants who are non-vigorous has not been recommended since the introduction of the 2015 Neonatal Resuscitation Program. Finally, some of the studies included in the analysis had small sample sizes. Further, well-designed RCTs with larger sample sizes are therefore needed to validate the potential benefits of gastric suction or gastric lavage in neonates. Overall, although the findings of this study were promising, more research is needed to understand the generalizability and effectiveness of the interventions in different populations and settings. There are also no studies investigating the effects of these procedures on non-vigorous infants or evaluating the efficacy of these procedures in non-vigorous infants with or without MSAF.

## Conclusions

Gastric suction or gastric lavage may be advantageous for newborns at birth. Vigorous infants with MSAF appear to have fewer GI symptoms after undergoing gastric lavage, but gastric suction may not provide any additional benefits beyond standard care in normal newborns. In addition, these procedures may delay breastfeeding. The risks and advantages of these procedures therefore need to be weighed carefully, and they may only be needed in high-risk babies who are predisposed to GI discomfort.

## Supporting information

S1 Table Baseline maternal and neonatal characteristics in the included studies. (DOCX)

S2 Table Summarized results of the included studies categorized by outcomes. (DOCX)

S1 Fig Risk-of-bias. Summary of the included studies using the Revised Cochrane Risk-of-Bias Tool for randomized trials (RoB 2).
(DOCX)

S2 Fig Funnel plots. Gastrointestinal symptoms in all neonates receiving gastric suction or gastric lavage.
(DOCX)

S1 Checklist PRISMA 2020 main checklist. (PDF)
