# Supplementary material for: Benefits and harms of gastric suction or lavage at birth for gastrointestinal outcomes: A systematic review and meta-analysis
Source: PLoS One. 2023 Jul 13;18(7):e0288398. doi: 10.1371/journal.pone.0288398 (PMC10343101; doi:10.1371/journal.pone.0288398)
Supplement: S1 Table — (DOCX) [file pone.0288398.s001.docx]

**S1 Table. Baseline maternal and neonatal characteristics in the included studies**

| **Study** | **Gastric suction/gastric lavage** | **Control** |
| --- | --- | --- |
| **Widström et al. 1987 [5]** | **n=11** | **n=10** |
| Maternal age, mean (SD), years | NR | NR |
| Non-NSVD (C-section); n (%) | NR | NR |
| Gestational age, mean (SD), weeks | 40.4 (1.33) * | 40.2 (1.26) * |
| Birth weight, mean (SD), g | 3694 (99.6) * | 3742 (101.1) * |
| Male; n (%) | 3 (27.3) | 2 (20) |
| Apgar score ≥8 at 1 min; n (%) | 11 (100) | 10 (100) |
| **Narchi & Kulaylat 1999 [7]** | **n=227** | **n=275** |
| Maternal age, mean (SD), years | NR | NR |
| Non-NSVD (C-section); n (%) | NR | NR |
| Gestational age, mean (SD), weeks | NR | NR |
| Birth weight, mean (SD), g | NR | NR |
| Male; n (%) | NR | NR |
| Thick meconium; n (%) | NR | NR |
| **Cuello-Garcia et al. 2005 [2]** | **n=63** | **n=61** |
| Maternal age, mean (SD), years | 26.3 (5.5) | 24.8 (5.7) |
| Non-NSVD (C-section); n (%) | 27 (43) | 15 (25) |
| Gestational age, mean (SD), weeks | 39.8 (1) | 39.8 (1.1) |
| Birth weight, mean (SD), g | 3264 (363) | 3193 (328) |
| Male; n (%) | 31 (49.2) | 18 (29.5) |
| **Kiremitci et al. 2011 [1]** | **n=155** | **n=154** |
| Maternal age, mean (SD), years | 30.3 (4.6) | 30.3 (5.1) |
| Non-NSVD (C-section); n (%) | 132 (85) | 131 (85) |
| Gestational age, mean (SD), weeks | 38.4 (0.9) | 38.4 (0.8) |
| Birth weight, mean (SD), g | 3394 (449) | 3321 (384) |
| Male; n (%) | 82 (53) | 84 (55) |
| **Ameta et al. 2013 [10]** | **n=124** | **n=120** |
| Maternal age, mean (SD), years | NR | NR |
| Non-NSVD (C-section or instruments); n (%) | 55 (44.4) | 50 (41.7) |
| Gestational age, mean (SD), weeks | 38.49 (1.68) | 38.64 (1.53) |
| Birth weight, mean (SD), g | 2753 (400) | 2731 (440) |
| Male; n (%) | 71 (57.3) | 70 (58.33) |
| Thick meconium; n (%) | 89 (71.77) | 90 (75) |
| **Singh et al. 2013 [11]** | **n=72** | **n=74** |
| Maternal age, mean (SD), years | NR | NR |
| Non-NSVD (C-section or instrument); n (%) | 38 (52.8) | 45 (60.8) |
| Gestational age, mean (SD), weeks | 38.3 (1.5) | 38.7 (1.3) |
| Birth weight, mean (SD), g | 2746 (450) | 2837 (423) |
| Male; n (%) | 39 (54.2) | 46 (62.2) |
| Apgar Score 1 min, mean (SD) | 8.9 (0.3) | 8.9 (0.4) |
| Small-for-gestational age | 6 (8.3) | 4 (5.4) |
| Thick meconium; n (%) | 38 (52.8) | 37 (50.0) |
| **Garg et al. 2014 [12]** | **n=165** | **n=153** |
| Maternal age, mean (SD), years | NR | NR |
| Non-NSVD (C-section); n (%) | 73 (44.2) | 60 (39.2) |
| Gestational age, n (%), weeks |  |  |
| 34–36 | 98 (59.4) | 69 (45.1) |
| 37–40 | 67 (40.6) | 84 (54.9) |
| Birth weight ≥2000 g, n (%) | 144 (87.3) | 134 (87.6) |
| Male; n (%) | 86 (52.1) | 80 (52.3) |
| Apgar score 1 min, mean (SD) | NR | NR |
| Thick meconium; n (%) | 40 (24.2) | 42 (27.5) |
| **Sharma et al. 2014 [13]** | **n=267** | **n=269** |
| Maternal age, mean (SD), years | NR | NR |
| Non-NSVD (C-section); n (%) | NR | NR |
| Gestational age, mean (SD), weeks | 38 (1.48) ** | 38 (1.48) ** |
| Birth weight, mean (SD), g | 2762 (499) | 2758 (448) |
| Male; n (%) | 150 (56.2) | 147 (54.6) |
| Apgar score 5 min, mean (SD) | 8 (1.48) ** | 8 (1.48) ** |
| Thick meconium; n (%) | 141 (52.8) | 147 (54.6) |
| **Shah et al. 2015 [14]** | **n=230** | **n=270** |
| Maternal age, mean (SD), years | NR | NR |
| Non-NSVD (C-section); n (%) | 105 (45.7) | 114 (42.2) |
| Male; n (%) | 125 (54.3) | 130 (48.1) |
| Gestational age, mean (SD), weeks | 37.3 (1.5) | 37.6 (1.3) |
| Birth weight, mean (SD), g | 2748 (450) | 2835 (425) |
| Apgar score 5 min, mean (SD) | 8 (1.48) ** | 8 (1.48) ** |
| Abdominal girth, mean (SD), cm | 25 (0.5) | 24 (1.0) |
| **Kumar et al. 2017 [15]** | **n=229** | **n=269** |
| Maternal age, mean (SD), years | NR | NR |
| Non-NSVD (C-section); n (%) | 104 (45.4) | 113 (42.0) |
| Male; n (%) | 125 (54.6) | 130 (48.3) |
| Gestational age, mean (SD), weeks | 37.2 (1.5) | 37.4 (1.4) |
| Birth weight, mean (SD), g | 2746 (445) | 2795 (420) |
| Apgar score 5 min, mean (SD) | 8 (1.48) ** | 8 (1.48) ** |
| Abdominal girth, mean (SD), cm | 24 (0.5) | 24 (1.0) |
| **Gidaganti et al. 2018 [16]** | **n=350** | **n=350** |
| Maternal age, mean (SD), years | NR | NR |
| Non-NSVD (C-section or instrument); n (%) | 134 (38.3) | 115 (32.9) |
| Gestational age, mean (SD), weeks | NR | NR |
| Preterm birth, n (%) | 94 (26.9) | 97 (27.7) |
| Birth weight, mean (SD), g | 2684 (398) | 2706 (398) |
| Male; n (%) | 182 (52.0) | 193 (55.1) |
| Apgar score 1 min, mean (SD) | 8.90 (0.30) | 8.90 (0.30) |
| Apgar score 5 min, mean (SD) | 9.00 (0.30) | 9.00 (0.30) |
| Meconiumcrit >30%, n (%) | 101 (28.9) | 90 (25.7) |
| **Yadav et al. 2018 [17]** | **n=107** | **n=117** |
| Maternal age, mean (SD), years | NR | NR |
| Non-NSVD (C-section), n (%) | 22 (20.6) | 31 (26.5) |
| Gestational age, mean (SD), weeks | 38 (0.74) ** | 38 (0.74) ** |
| Birth weight, mean (SD), g | 2948 (450) | 3035 (425) |
| Male; n (%) | 56 (52.3) | 61 (52.1) |
| Apgar score 5 min, mean (SD) | 8 (0.74) ** | 8 (0.74) ** |

**Abbreviations:** C-section, cesarean section; NSVD, normal spontaneous vaginal delivery; NR, no report; SD, standard deviation

*Converted data from mean (standard error of mean) to mean (SD)

**Converted data from median (interquartile range) to mean (SD)
