# Supplementary material for: Benefits and harms of gastric suction or lavage at birth for gastrointestinal outcomes: A systematic review and meta-analysis
Source: PLoS One. 2023 Jul 13;18(7):e0288398. doi: 10.1371/journal.pone.0288398 (PMC10343101; doi:10.1371/journal.pone.0288398)
Supplement: S2 Table — (DOCX) [file pone.0288398.s002.docx]

**S2 Table. Summarized results of the included studies categorized by outcomes**

| **Author (year)** | | **Intervention** | **Gastric suction/Gastric lavage** | | | | | | | **Control** | | | | | **Risk Ratio  (95% CI)** |
| --- | --- | --- | --- | --- | --- | --- | --- | --- | --- | --- | --- | --- | --- | --- | --- |
|  |  |  | **Event/** | | | **Total** | | | | **Event** | | **Total** | | |  |
| **Benefits of gastric suction or gastric lavage** | | | | | | | | | | | | | | | |
| **Gastrointestinal symptoms (retching, vomiting)** | | | | | | | | | | | | | | | |
| **All neonates** | | | | | | | | | | | | | | | |
| **Widström et al. 1987 [5]** | | Gastric suction | 9 | | | 11 | | | | 7 | | 10 | | | 1.17 [0.71, 1.91] |
| **Narchi & Kulaylat 1999 [7]** | | Gastric lavage | 0 | | | 227 | | | | 13 | | 275 | | | 0.04 [0.00, 0.75] |
| **Cuello-Garcia et al. 2005 [2]** | | Gastric suction | 13 | | | 63 | | | | 15 | | 61 | | | 0.84 [0.44, 1.61] |
| **Kiremitci et al. 2011 [1]** | | Gastric suction | 10 | | | 155 | | | | 16 | | 154 | | | 0.62 [0.29, 1.33] |
| **Ameta et al. 2013 [10]** | | Gastric lavage | 12 | | | 124 | | | | 16 | | 120 | | | 0.73 [0.36, 1.47] |
| **Singh et al. 2013 [11]** | | Gastric lavage | 22 | | | 72 | | | | 20 | | 74 | | | 1.13 [0.68, 1.89] |
| **Garg et al. 2014 [12]** | | Gastric lavage | 16 | | | 165 | | | | 21 | | 153 | | | 0.71 [0.38, 1.30] |
| **Sharma et al. 2014 [13]** | | Gastric lavage | 18 | | | 267 | | | | 29 | | 269 | | | 0.63 [0.36, 1.10] |
| **Shah et al. 2015 [14]** | | Gastric lavage | 20 | | | 230 | | | | 31 | | 270 | | | 0.76 [0.44, 1.29] |
| **Kumar et al. 2017 [15]** | | Gastric lavage | 20 | | | 229 | | | | 31 | | 269 | | | 0.76 [0.44, 1.29] |
| **Gidaganti et al. 2018 [16]** | | Gastric lavage | 82 | | | 350 | | | | 125 | | 350 | | | 0.66 [0.52, 0.83] |
| **Yadav et al. 2018 [17]** | | Gastric lavage | 5 | | | 107 | | | | 11 | | 117 | | | 0.50 [0.18, 1.38] |
| Subtotal (95% CI)  Heterogeneity: Tau² = 0.01; χ² = 12.75, df = 11 (P = 0.31); I² = 14%  Test for overall effect: Z = 3.25 (P = 0.001) | | | | | | | | | | | | | | | 0.75 [0.63, 0.89] |
| **No meconium-stained amniotic fluid neonates** | | | | | | | | | | | | | | | |
| **Widström et al. 1987 [5]** | | Gastric suction | 9 | | | 11 | | | | 7 | | 10 | | | 1.17 [0.71, 1.91] |
| **Cuello-Garcia et al. 2005 [2]** | | Gastric suction | 13 | | | 63 | | | | 15 | | 61 | | | 0.84 [0.44, 1.61] |
| **Kiremitci et al. 2011 [1]** | | Gastric suction | 10 | | | 155 | | | | 16 | | 154 | | | 0.62 [0.29, 1.33] |
| Subtotal (95% CI)  Heterogeneity: Tau² = 0.03; χ² = 2.60, df = 2 (P = 0.27); I² = 23%  Test for overall effect: Z = 0.46 (P = 0.65) | | | | | | | | | | | | | | | 0.91 [0.61, 1.37] |
| **Meconium-stained amniotic fluid neonates** | | | | | | | | | | | | | | | |
| **Narchi& Kulaylat 1999 [7]** | | Gastric lavage | 0 | | | 227 | | | | 13 | | 275 | | | 0.04 [0.00, 0.75] |
| **Ameta et al. 2013 [10]** | | Gastric lavage | 12 | | | 124 | | | | 16 | | 120 | | | 0.73 [0.36, 1.47] |
| **Singh et al. 2013 [11]** | | Gastric lavage | 22 | | | 72 | | | | 20 | | 74 | | | 1.13 [0.68, 1.89] |
| **Garg et al. 2014 [12]** | | Gastric lavage | 16 | | | 165 | | | | 21 | | 153 | | | 0.71 [0.38, 1.30] |
| **Sharma et al. 2014 [13]** | | Gastric lavage | 18 | | | 267 | | | | 29 | | 269 | | | 0.63 [0.36, 1.10] |
| **Shah et al. 2015 [14]** | | Gastric lavage | 20 | | | 230 | | | | 31 | | 270 | | | 0.76 [0.44, 1.29] |
| **Kumar et al. 2017 [15]** | | Gastric lavage | 20 | | | 229 | | | | 31 | | 269 | | | 0.76 [0.44, 1.29] |
| **Gidaganti et al. 2018 [16]** | | Gastric lavage | 82 | | | 350 | | | | 125 | | 350 | | | 0.66 [0.52, 0.83] |
| **Yadav et al. 2018 [17]** | | Gastric lavage | 5 | | | 107 | | | | 11 | | 117 | | | 0.50 [0.18, 1.38] |
| Subtotal (95% CI)  Heterogeneity: Tau² = 0.00; χ² = 8.25, df = 8 (P = 0.41); I² = 3%  Test for overall effect: Z = 3.98 (P < 0.0001) | | | | | | | | | | | | | | | 0.71 [0.60, 0.84] |
| **Feeding intolerance** | | | | | | | | | | | | | | | |
| **Thick-Meconium-stained amniotic fluid neonates** | | | | | | | | | | | | | | | |
| **Ameta et al. 2013 [10]** | | Gastric lavage | 10 | | | 89 | | | | 13 | | 90 | | | 0.78 [0.36, 1.68] |
| **Garg et al. 2014 [12]** | | Gastric lavage | 7 | | | 40 | | | | 8 | | 42 | | | 0.92 [0.37, 2.30] |
| Subtotal (95% CI)  Heterogeneity: Tau² = 0.00; χ² = 0.07, df = 1 (P = 0.79); I² = 0%  Test for overall effect: Z = 0.62 (P = 0.54) | | | | | | | | | | | | | | | 0.83 [0.46, 1.50] |
| **Thin-Meconium-stained amniotic fluid neonates** | | | | | | | | | | | | | | | |
| **Ameta et al. 2013 [10]** | | Gastric lavage | 2 | | | 35 | | | | 3 | | 30 | | | 0.57 [0.10, 3.20] |
| **Garg et al. 2014 [12]** | | Gastric lavage | 9 | | | 125 | | | | 13 | | 111 | | | 0.61 [0.27, 1.38] |
| Subtotal (95% CI)  Heterogeneity: Tau² = 0.00; χ² = 0.01, df = 1 (P = 0.94); I² = 0%  Test for overall effect: Z = 1.34 (P = 0.18) | | | | | | | | | | | | | | | 0.61 [0.29, 1.26] |
| **Secondary pulmonary aspiration neonates** | | | | | | | | | | | | | | | |
| **Narchi & Kulaylat 1999 [7]** | | Gastric lavage | 0 | | | 227 | | | | 0 | | 275 | | | Not estimable |
| **Ameta et al. 2013 [10]** | | Gastric lavage | 0 | | | 124 | | | | 0 | | 120 | | | Not estimable |
| **Singh et al. 2013 [11]** | | Gastric lavage | 0 | | | 72 | | | | 0 | | 74 | | | Not estimable |
| **Garg et al.2014 [12]** | | Gastric lavage | 0 | | | 165 | | | | 0 | | 153 | | | Not estimable |
| **Sharma et al. 2014 [13]** | | Gastric lavage | 0 | | | 267 | | | | 0 | | 269 | | | Not estimable |
| **Gidaganti et al. 2018 [16]** | | Gastric lavage | 5 | | | 350 | | | | 8 | | 350 | | | 0.63 [0.21, 1.89] |
| **Yadav et al. 2018 [17]** | | Gastric lavage | 0 | | | 107 | | | | 0 | | 117 | | | Not estimable |
| Subtotal (95% CI)  Heterogeneity: Not applicable  Test for overall effect: Z = 0.83 (P = 0.41) | | | | | | | | | | | | | | | 0.63 [0.21, 1.89] |
| **Author (year)** | **Intervention** | | **Gastric suction/ gastric lavage** | | | | | | **Control** | | | | | | **Mean difference (95% CI)** |
|  |  |  | **Mean** | **SD** | | | **N** | | **Mean** | | **SD** | | **N** | |  |
| **Time to initiate breastfeeding (min)** | | | | | | | | | | | | | | | |
| **Widström et al. 1987 [5]** | | Gastric suction | 62 | | 19.92* | | | 11 | | 55 | 12.64* | | | 10 | 7.00 [−7.14, 21.14] |
| **Kiremitci et al. 2011 [1]** | | Gastric suction | 61 | | 20.9 | | | 155 | | 60 | 18.1 | | | 154 | 1.00 [−3.36, 5.36] |
| **Sharma et al. 2014 [13]** | | Gastric lavage | 25 | | 9 | | | 267 | | 22 | 7 | | | 269 | 3.00 [1.63, 4.37] |
| Subtotal (95% CI)  Heterogeneity: Tau² = 0.00; χ² = 1.07, df = 2 (P = 0.59); I² = 0%  Test for overall effect: Z = 4.31 (P < 0.0001) | | | | | | | | | | | | | | | 2.86 [1.56, 4.15] |

**Abbreviations:** CI, confidence interval; SD, standard deviation

* Converted data from mean (standard error of mean) to mean (SD)
