# Supplementary material for: Benefits and harms of gastric suction or lavage at birth for gastrointestinal outcomes: A systematic review and meta-analysis
Source: PLoS One. 2023 Jul 13;18(7):e0288398. doi: 10.1371/journal.pone.0288398 (PMC10343101; doi:10.1371/journal.pone.0288398)

**S1 Fig. Risk-of-bias**. Summary of the included studies using the Revised Cochrane Risk-of-Bias Tool for randomized trials (RoB 2)


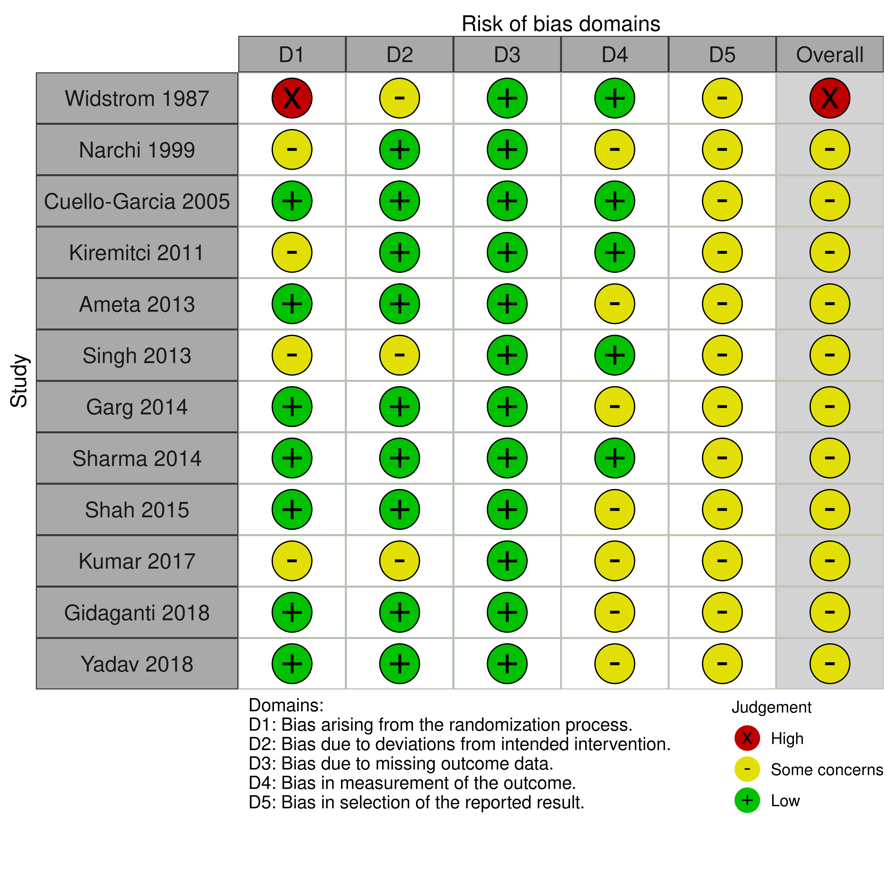

Supplement: S1 Fig — Summary of the included studies using the Revised Cochrane Risk-of-Bias Tool for randomized trials (RoB 2). (DOCX) [file pone.0288398.s003.docx]
