# Supplementary material for: Benefits and harms of gastric suction or lavage at birth for gastrointestinal outcomes: A systematic review and meta-analysis
Source: PLoS One. 2023 Jul 13;18(7):e0288398. doi: 10.1371/journal.pone.0288398 (PMC10343101; doi:10.1371/journal.pone.0288398)
Supplement: S2 Fig — Gastrointestinal symptoms in all neonates receiving gastric suction or gastric lavage. (DOCX) [file pone.0288398.s004.docx]

**S2 Fig. Funnel plots**. Gastrointestinal symptoms in all neonates receiving gastric suction or gastric lavage.

**
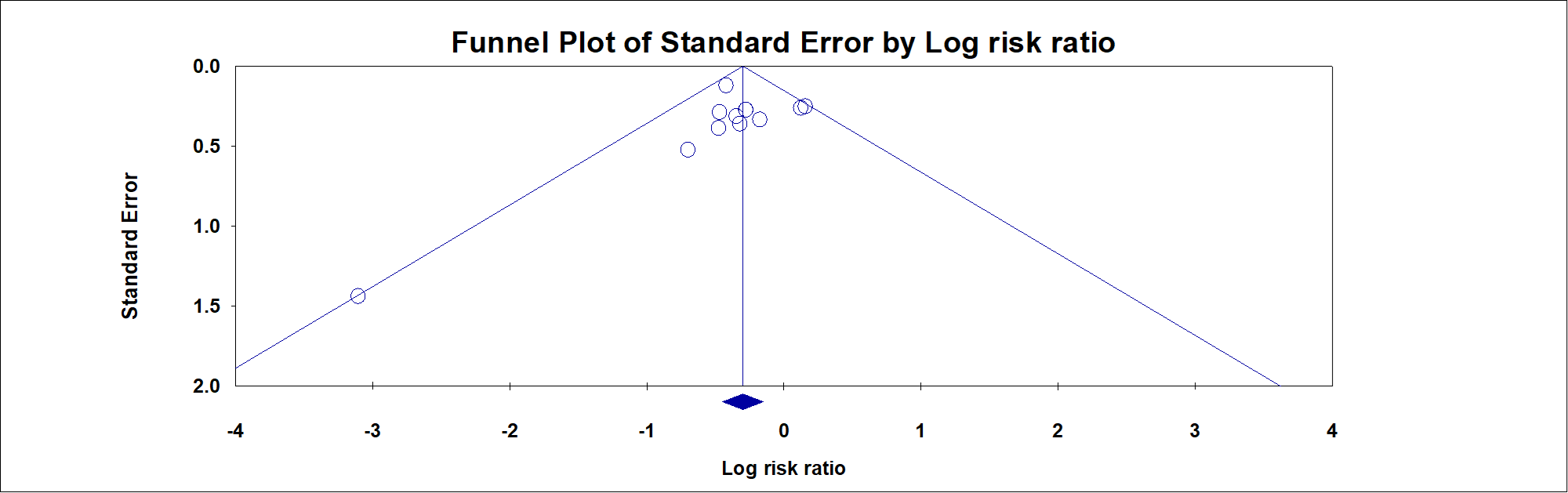
**
